# Genetically Encoded FRET-Sensor Based on Terbium Chelate and Red Fluorescent Protein for Detection of Caspase-3 Activity

**DOI:** 10.3390/ijms160716642

**Published:** 2015-07-22

**Authors:** Alexander S. Goryashchenko, Maria G. Khrenova, Anna A. Bochkova, Tatiana V. Ivashina, Leonid M. Vinokurov, Alexander P. Savitsky

**Affiliations:** 1 A. N. Bach Institute of Biochemistry, Russian Academy of Sciences, 119071 Moscow, Russia; E-Mail: asgoryash@inbi.ras.ru; 2 M. V. Lomonosov Moscow State University, Department of Chemistry, 119991 Moscow, Russia; E-Mails: wasabiko13@gmail.com (M.G.K.); bochka567@gmail.com (A.A.B.); 3 Skryabin Institute of Biochemistry and Physiology of Microorganisms, Russian Academy of Sciences, 142290 Pushchino, Russia; E-Mail: ivashina@ibpm.pushchino.ru; 4 Branch of Shemyakin and Ovchinnikov Institute of Bioorganic Chemistry, Russian Academy of Sciences, 142290 Pushchino, Russia; E-Mail: levino@bibch.ru

**Keywords:** FRET-sensor, caspase-3, terbium, fluorescent proteins, apoptosis

## Abstract

This article describes the genetically encoded caspase-3 FRET-sensor based on the terbium-binding peptide, cleavable linker with caspase-3 recognition site, and red fluorescent protein TagRFP. The engineered construction performs two induction-resonance energy transfer processes: from tryptophan of the terbium-binding peptide to Tb^3+^ and from sensitized Tb^3+^ to acceptor—the chromophore of TagRFP. Long-lived terbium-sensitized emission (microseconds), pulse excitation source, and time-resolved detection were utilized to eliminate directly excited TagRFP fluorescence and background cellular autofluorescence, which lasts a fraction of nanosecond, and thus to improve sensitivity of analyses. Furthermore the technique facilitates selective detection of fluorescence, induced by uncleaved acceptor emission. For the first time it was shown that fluorescence resonance energy transfer between sensitized terbium and TagRFP in the engineered construction can be studied via detection of microsecond TagRFP fluorescence intensities. The lifetime and distance distribution between donor and acceptor were calculated using molecular dynamics simulation. Using this data, quantum yield of terbium ions with binding peptide was estimated.

## 1. Introduction

Nowadays biosensors based on the Förster resonance energy transfer (FRET) phenomenon are widely used to study the enzymatic activity in living cells [1]. Change of the FRET efficiency allows direct monitoring of enzymatic activity, protein-protein interactions, conformational changes in proteins, *etc.* [2,3,4,5].

In FRET-sensors, energy transfer represents a dynamic type of fluorescence quenching of the donor and simultaneous increase of the acceptor fluorescence, respectively. Therefore, FRET can be detected by (1) a decrease of the donor’s fluorescence intensity; (2) an increase of the acceptor fluorescence intensity; (3) in dual channel mode measurements (at two wavelengths)—by change of the ratio of donor and acceptor fluorescence intensities. All these measurements can be carried out not only in the stationary mode (fluorescence intensity), but also in time-resolved mode, because FRET is also characterized by a decrease of the donor fluorescence lifetime and increase of the appeared acceptor fluorescence lifetime [4,5,6,7]. FRET efficiency measurement using fluorescence lifetime change is the most accurate and reliable method, since the fluorescence lifetime is independent of concentration of fluorophores, photobleaching, changes in the intensity of the excitation light, and light scattering [4,5].

Use of fluorescent complexes of lanthanides, which have a microsecond fluorescence, as donors in FRET-pair, and spectroscopy with time delay allows us to eliminate short-lived background signals associated with the autofluorescence of biomolecules and scattered light [3,5,8]. In addition, measurements with time delay detect only sensitized fluorescence of the acceptor. It solves the problem of simultaneous fluorescence excitation of the donor and acceptor thereby increasing the dynamic range of measurements and the accuracy of the FRET efficiency determination [5].

Lanthanide-based FREТ sensors were used to study interactions between proteins [9] and subunits of oligomeric proteins [10,11,12]; to detect the conformational changes induced by the binding of activators and repressors [13], DNA hybridization in solutions, and cell extracts and in cell culture [14,15,16]; to analyze the enzymatic activity [17,18,19,20,21,22,23], for example the catalytic activity of key enzymes of apoptosis—caspases.

Apoptosis is a genetically regulated process of cell death, which contributes to normal functioning of the organism through the destruction of useless, diseased, and mutated cells which are potentially dangerous and the ones which have finished their life cycle. Dysregulation of apoptosis underlies many neurodegenerative, neoplastic, and autoimmune diseases [24].

In most cases apoptosis is associated with the activation of caspases—a family of proteolytic enzymes that specifically cleave proteins at the N-terminus of aspartic acid residues. Caspase-3 is a main effector caspase uniting mitochondrial and receptor pathways of apoptosis. In addition, it is expressed in all tissues and involved in almost all the currently known cycles of amplification [25]. Therefore, the caspase-3 activity is a useful marker for determining the aggressiveness of pathological processes and evaluation of efficiency of apoptosis-inducing drugs.

**Figure 1:**
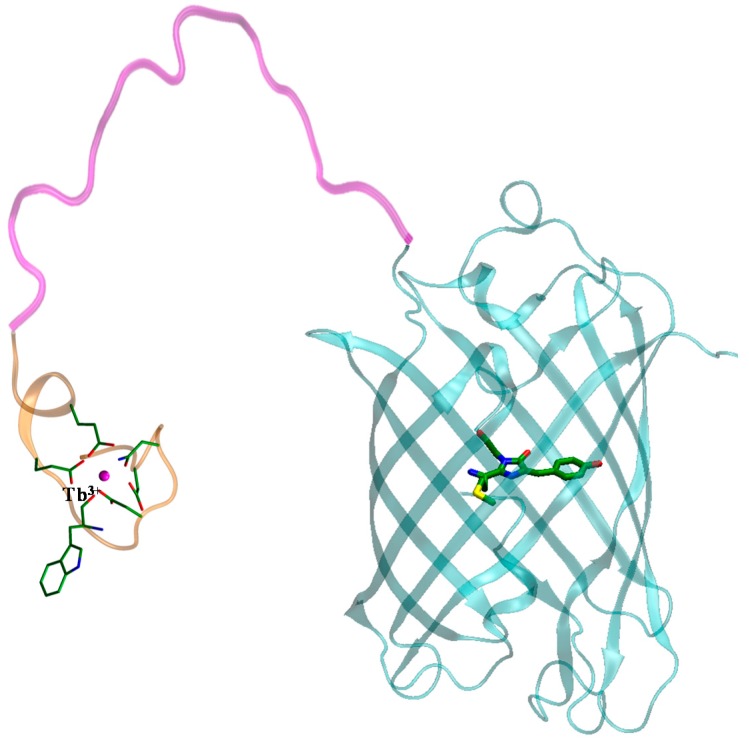
Structure of Tb^3+^-TBP-19-TagRFP sensor. Carbon atoms are shown in green, nitrogen atoms are shown in blue, and sulphur atom is shown in yellow.

Our laboratory has developed a genetically encoded FRET sensor for caspase-3 Tb^3+^-TBP-19-TagRFP [26,27], which consists of the complex of Tb^3+^ with terbium-binding peptide YIDTNNDGWYEGDELLA [28] as a donor, flexible linker VDGGSGGDEVDGWGGSGLD [29] with the caspase-3 cleavage site DEVD [30], which was used earlier in the FRET-pair with a non-fluorescent acceptor [31], and red fluorescent protein TagRFP [26,32] as an acceptor. There are two donor-acceptor pairs in the resulting structure, which transfer energy using the fluorescence resonance mechanism. Initially, energy transfers from the tryptophan in the terbium-binding peptide, which sensitizes terbium ion, and then from the terbium ion to the TagRFP chromophore (Figure 1).

The fluorescence of the sensor is in the orange spectral region that can reduce the contribution of background fluorescence in the total intensity. In addition, unlike previously developed lanthanide sensors, in which cryptand-lanthanide complex was a donor, and allophycocyanin or synthetic fluorophores as acceptor [15,16], the Tb^3+^-TBP-19-TagRFP sensor is genetically encoded and requires only the additional introduction of terbium ions, which on one hand allows for highly standardized samples for *in vitro* measurements, and on the other hand in the long term makes its use in experiments on living cells possible.

## 2. Results and Discussion

### 2.1. Molecular Dynamics Simulations

**Figure 2:**
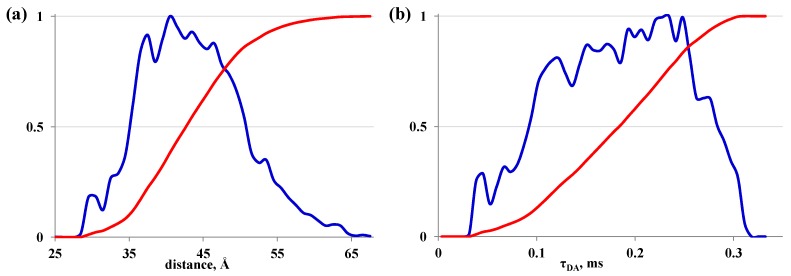
(**a**) Distance distribution between donor and acceptor (blue curve) and corresponding cumulative probability (red curve); (**b**) lifetime distribution *τ_DA_* (blue curve) and corresponding cumulative probability (red curve).

We performed 200 ns molecular dynamics simulation of the linker connecting energy donor and acceptor in a water box to study its conformational dynamics. MD simulation demonstrates that the linker is rather flexible resulting in a wide distribution of distances between donor and acceptor (Figure 2a). FRET efficiency *E* and energy transfer rate constant *k_T_* as well as lifetime of donor in the presence of acceptor *τ_DA_* were calculated on the fly for each frame of MD trajectory (Figure 2b). Lifetime distribution *τ_DA_* is broad and corresponds to the mean value 0.19 ms that is in good agreement with the experimentally observed value. FRET efficiency calculated as a mean value from all frames is 43% (compared with 44% from experiment).

### 2.2. Determination of Oligomeric State of the Protein

Using the dynamic light scattering technique, the effective diffusion radii of the sensor sample and their corresponding molecular masses were obtained. Sample protein TBP-19-TagRFP contains molecules with a radius of 3.5 and 2.6 nm, which in the spherical approximation correspond to the molecular mass of 62 and 33 kDa, respectively.

The oligomeric state of the protein TBP-19-TagRFP was also determined using gel-filtration chromatography. The target protein was collected by absorption at 560 nm. In the chromatogram there were two peaks, first one corresponded to the molecular mass of 60 kDa, the second—to 28 kDa.

Molecular weight of the protein, calculated from the amino acid sequence, is 30.5 kDa. The data obtained in two independent experiments gave the same results—the protein TBP-19-TagRFP is a mixture of dimeric and monomeric forms.

### 2.3. Fluorimetric Titration of the TBP-19-TagRFP Protein Using Tb(NO_3_)_3_ Solution

**Figure 3:**
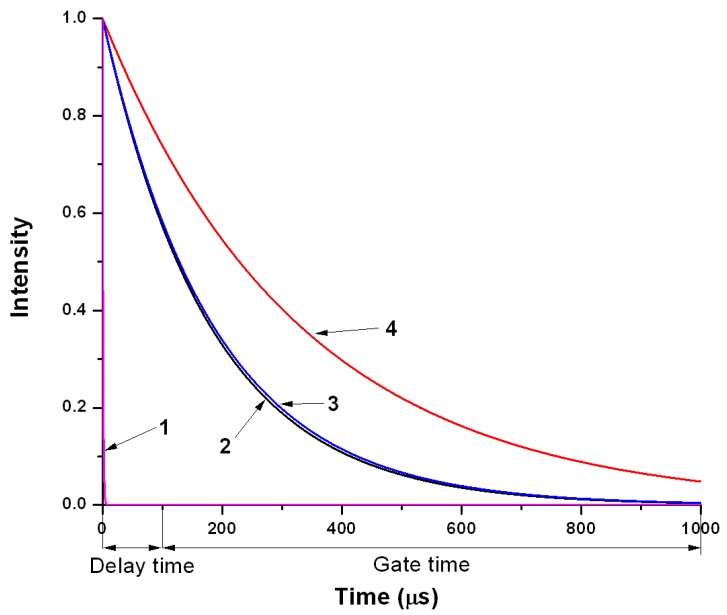
The principle of fluorescence spectroscopy with a time delay. (1) is the light flash; (2) is the fluorescence of the donor in the presence of acceptor; (3) is the sensitized fluorescence of acceptor; (4) is the fluorescence of the donor without the acceptor.

To measure the sensor signal, fluorimetric titration of terbium binding sites was performed. The time delay technique in such experiments (Figure 3) permits selective measurement of the fluorescence of the sensor without the influence of background autofluorescence and scattered light. In the limit of a short acceptor lifetime, gated measurement after a delay time of 100 µs the fluorescence of directly excited acceptor is negligible, while the acceptor emission resulting from energy transfer displays almost the same lifetime as the quenched donor [7].

Registration of spectra was performed with a time delay of 100 μs, allowing to exclude the contribution of the direct excitation of the acceptor to the total intensity, and to measure only the fluorescence of the acceptor due to the energy transfer. In addition, the time delay cut off the short-lived background fluorescence [26].

**Figure 4:**
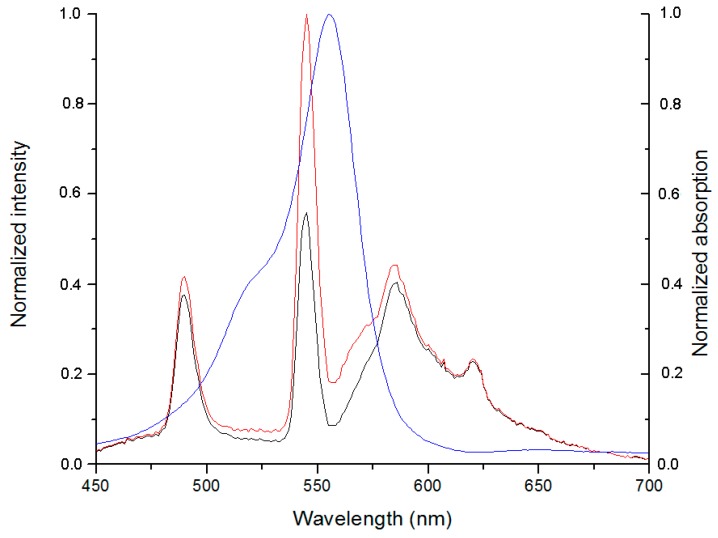
Overlay of absorption and fluorescence spectra of the Tb^3+^-TBP-19-TagRFP sensor. The absorption spectrum is shown by the blue line, the original spectrum of the fluorescence is shown by the black line, and the corrected fluorescence spectrum is shown by the red line.

However, at high concentration of the sensor its fluorescence intensity is lower than expected because of the reabsorption effect, which leads to distortion of the short-wave part of the TagRFP emission spectrum, where absorption of the sample is particularly high (Figure 4):

If the fluorescence path is considered from the center of the cuvette, the corrected fluorescence intensity is determined by the ratio (1):

(1) $$I_{\text{corr}}=I\times 10^{\frac{D}{2}}\;$$

where *I*_corr_ is the corrected fluorescence intensity, *I* is measured fluorescence intensity, *D* is the optical density of the solution.

**Figure 5:**
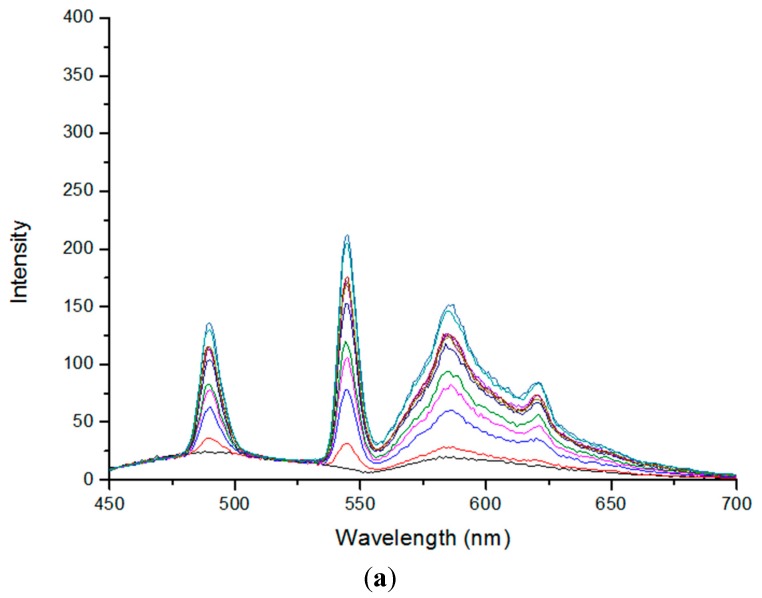

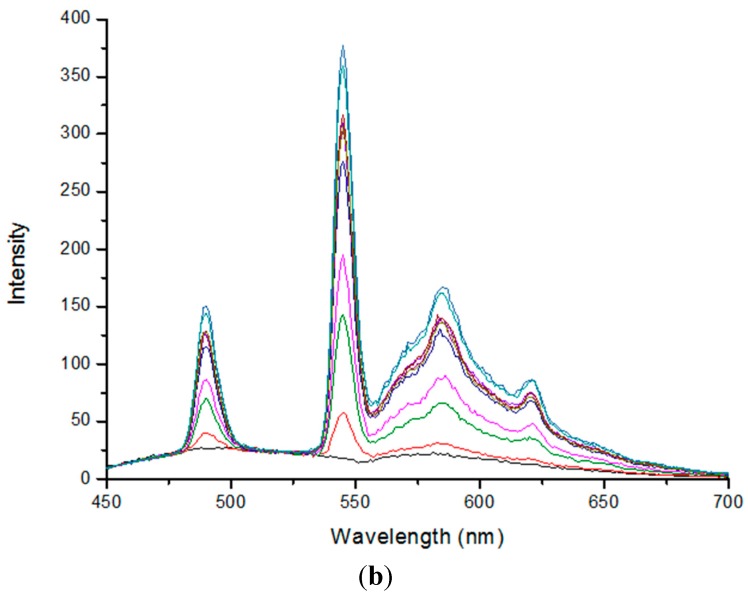
Fluorescence spectra of the Tb^3+^-TBP-19-TagRFP sensor before (**a**) and after (**b**) the correction for reabsorption. Measurements were performed using 280 nm excitation wavelength and a time delay of 100 μs, gate 1 ms in the phosphorescence mode. Spectra are arranged one above the other with the terbium concentration increase from 1 to 50 µM.

The results of such correction are shown in the Figure 5. The intensity of corrected spectra (Figure 5b) is higher than initial one (Figure 5a).

**Figure 6:**
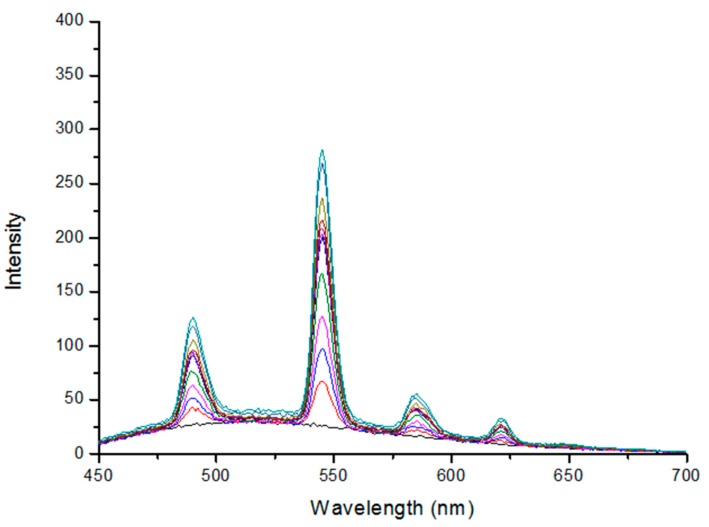
Fluorescence spectra of the Tb^3+^-TBP-19-TagRFP sensor with photobleached TagRFP chromophore. Measurements were performed using 280 nm excitation wavelength and a time delay of 100 μs, gate 1 ms in the phosphorescence mode. Spectra are arranged one above the other with the terbium concentration increase from 1 to 50 µM.

In order to calculate the TagRFP fluorescence without the contribution of terbium fluorescence, it was necessary to carry out the titration of terbium-binding peptide linked to the TagRFP protein with photobleached chromophore using Tb(NO_3_)_3_ solution as described in [26]. The results of that titration are shown in Figure 6.

**Figure 7:**
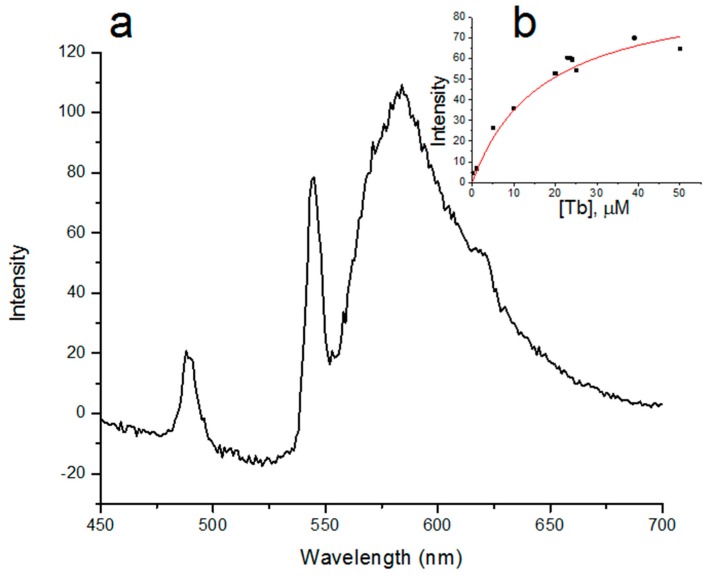
(**a**) Spectrum of the sensitized fluorescence of TagRFP in the Tb^3+^-TBP-19-TagRFP sensor; (**b**) The dependence of sensitized fluorescence intensity of TagRFP, which was calculated by subtraction of intensity of the photobleached sample from the intensity of the non-bleached sample corrected for reabsorption, on the Tb concentration at 606 nm.

The fluorescence intensity of TagRFP (Figure 7a) was calculated by subtraction of intensity of the photobleached sample (Figure 6), which lacks the signal from the TagRFP chromophore, from the intensity of the initial non-bleached sample (Figure 5).

As a result, we showed that there is an energy transfer in the Tb^3+^-TBP-19-TagRFP sensor from the terbium ion to the TagRFP chromophore. For the first time we obtained the spectrum of the sensitized TagRFP fluorescence with time delay for the previously described sensor [26,27]. Using the titration curve of TBP-19-TagRFP at 606 nm (Figure 7b), which was obtained by subtraction of intensity of the photobleached sample from the intensity of the corrected for reabsorption non-bleached sample, the dissociation constant of Tb^3+^ and TBP-19-TagRFP complex was calculated. We performed nonlinear curve fit of the titration curve in Origin 9.0.0 software using Equation (2):

(2) $$I=\frac{I_{\max}∙\text{[Tb]}}{K_{d}+\text{[Tb]}}$$

where *I* is measured fluorescence intensity, *I*_max_ (which was the fitting parameter) is the maximum fluorescence intensity at the saturating concentration, [Tb] is the Tb^3+^ concentration and *K*_d_ is the dissociation constant.

The saturating concentration was found as the intersection of tangents to the linear parts of the fitting curve (Figure 7b) and is equal to 24.2 µM. I_max_ is 95 ± 16 and the calculated *K*_d_ value is 17 ± 7 µM.

## 3. Experimental Section

### 3.1. Molecular Dynamics Simulation

We applied the NAMD program [33] to perform molecular dynamics simulations. We manually constructed oligopeptide representing linker between donor and acceptor in the inline conformation. The structure was protonated according to the basic principles of valences. Linker VDGGSGGDEVDGWGGSGLD was solvated in a rectangular water box 69 × 53 × 48 Å^3^ and neutralized by adding 0.2 M NaCl. Protein was described with CHARMM27 force field and water molecules with TIP3P model [34,35]. All long range electrostatic interactions were computed by using the particle mesh Ewald method [36]. Constant temperature MD simulations were performed for the NPT ensemble at 300 K using the Langevin thermostat. Following a 2000 step energy minimization, the MD simulations were carried out with a 2 fs integration time step and the total length of 200 ns. Rigid bonds approximation was imposed for the whole system. The VMD program was used for the visualization of the results [37].

### 3.2. Calculation of FRET Parameters

All FRET parameters, *i.e.*, efficiency E, rate constant of energy transfer *k_T_*, and lifetime of donor in the presence of acceptor *τ_DA_* were calculated according to the conventional formulae of FRET theory [7] on the fly for each MD frame. The procedure was as follows. The distance between donor and acceptor was extracted from each frame and *k_T_* was calculated as:

(3) $$k_{T}\left(r\right)=\frac{Q_{D}{\text{κ}}^{2}}{{\text{τ}}_{D}r^{6}}\left(\frac{9000\left(ln10\right)}{128{\text{π}}^{5}Nn^{4}}\right)J\left(\text{λ}\right)$$

where *r*, *J*(λ), *N* and *n* are distance between donor and acceptor, overlap integral, Avogadro’s number, and refractive index, respectively. In assumption of a freely rotational donor and acceptor, we used orientation factor κ^2^ equal to 2/3. FRET efficiency was calculated for each frame from the corresponding *k_T_* value as:

(4) $$E=\frac{k_{T}}{{\text{τ}}_{D}^{-1}+k_{T}}$$

The lifetime of donor in the presence of acceptor *τ_DA_* was calculated as:

(5) $${\text{τ}}_{DA}={\text{τ}}_{D}\left(1-E\right)$$

Quantum yield of donor *Q_D_* value was estimated from the experimental lifetime of the donor without acceptor τ*_D_* = 0.33 ms [26] and radiative lifetime of Tb^3+^ τ*_D_*_,*R*_ = 3.5 ms [38] as 0.094. Overlap integral was calculated from the experimental data and equals to 3.22591 × 10^15^ М^−1^·cm^−1^·nm^4^.

### 3.3. Determination of Oligomeric State of the Protein

Determination of oligomeric state was carried out using the hydrodynamic radii data obtained on DynaPro Titan (Wyatt Technology, Goleta, CA, USA) and Dynamics 6.6.7.9 software (Wyatt Technology, Goleta, CA, USA). The sample protein was in 20 mM·Tris-HCl, pH 7.5 buffer at 25 °C, the intensity of laser irradiation was set to 100%. For the interpretation of the results we used a Globular protein model.

The oligomeric state of the sensor was also determined using the molecular mass values obtained by gel-filtration chromatography. The separation of components was performed on the Superdex 200 10/300 GL column using ÄKTA Purifier (GE Healthcare, Little Chalfont, UK). The elution buffer was 20 mM Tris-HCl, pH 7.5. Registration of absorption was performed at two wavelengths—280 and 560 nm. The protein was collected by absorption at 560 nm.

### 3.4. Spectrophotometric Analysis

Absorption spectra were measured using Cary 300 (Varian, Palo Alto, CA, USA) spectrophotometer and 3 mm quartz microcuvette. Sample was in 20 mM Tris-HCl, pH 7.5 buffer.

### 3.5. Spectrofluorimetric Analysis

All samples were in 20 mm Tris-HCl, pH 7.5 buffer, measurements were performed in a 3 mm quartz microcuvette. 23.4 µM sample protein was titrated using the terbium nitrate (III) in 0.01 M HCl (AppliChem, Darmstadt, Germany) to a final concentration of 50 μM. Cary Eclipse spectrofluorometer (Varian, Palo Alto, CA, USA) was used for fluorescence measurements. Measurements were performed using 280 nm excitation wavelength and a time delay of 100 μs, gate 1 ms in the phosphorescence mode, excitation and emission slits were set to 20 and 5 nm, respectively. TagRFP photobleaching was carried out using a 532 nm laser with 105 mW power. Sample irradiation was continued until TagRFP fluorescence reached zero.

## 4. Conclusions

For the first time we have detected sensitized TagRFP fluorescence using microsecond time gated technique. This approach allows to solve the problem of direct acceptor excitation due to spectral cross-talk between donor and acceptor. In Ref. [26] FRET efficiency of 44% was detected by change of donor (terbium ion) fluorescence lifetime from 0.33 ms for photobleached form of Tb^3+^-TBP-19-TagRFP to 0.18 ms for initial form. This method also allows the exclusion of signal cross-talk, but fluorescence lifetime of terbium ion can vary because of changes in its coordination sphere [39], decreasing the accuracy of the results. Direct detection of sensitized TagRFP fluorescence using time gated approach allows us to get rid of both problems mentioned above, because we only measure the signal that is connected to FRET, while the background fluorescence and signal from directly excited TagRFP were already gone to zero during the delay time. We calculated the K_d_ value of the Tb^3+^ and TBP-19-TagRFP complex and found it to be 17 ± 7 µM.

The molecular dynamics modeling allowed us to calculate the distribution of possible distances between the donor and acceptor in Tb^3+^-TBP-19-TagRFP sensor. Calculated FRET efficiency is equal to 43% which in good agreement with the experimental value of 44%. 
